# A feasible CT feature to differentiate focal‐type autoimmune pancreatitis from pancreatic ductal adenocarcinoma

**DOI:** 10.1002/cam4.2526

**Published:** 2019-08-30

**Authors:** Chaobin He, Dailin Rong, Wanming Hu, Qian Cai, Qiuxia Yang, Yize Mao, Rong Zhang, Shengping Li, Yanchun Lv

**Affiliations:** ^1^ State Key Laboratory of Oncology in South China Department of Pancreatobiliary Surgery Collaborative Innovation Center for Cancer Medicine Sun Yat‐sen University Cancer Center Guangzhou China; ^2^ Department of Radiology The Third Affiliated Hospital Sun Yat‐sen University (SYSU) Guangzhou Guangdong China; ^3^ State Key Laboratory of Oncology in South China Department of Radiology Collaborative Innovation Center for Cancer Medicine Sun Yat‐sen University Cancer Center Guangzhou China; ^4^ State Key Laboratory of Oncology in South China Department of Pathology Collaborative Innovation Center for Cancer Medicine Sun Yat‐sen University Cancer Center Guangzhou China

**Keywords:** autoimmune pancreatitis, computed tomography, pancreatic ductal adenocarcinoma

## Abstract

**Background:**

To investigate whether the relative computed tomography (CT) value (rCT) of adjacent pancreatic parenchyma can distinguish focal‐type autoimmune pancreatitis (fAIP) from pancreatic ductal adenocarcinoma (PDAC).

**Methods:**

A total of 13 patients with fAIP and 20 patients with PDAC were included in this study. The rCT was calculated as the ratio of the CT value of adjacent pancreatic parenchyma to that of muscle. The diagnostic performance of rCT for discriminating fAIP from PDAC was evaluated using receiver operating characteristic (ROC) analysis.

**Results:**

Both fAIP and PDAC presented hyper‐fibrosis histologically and delayed enhancement on CT examination. Moreover, the pancreatic parenchyma of fAIP presented serious inflammation. The mean rCT of the parenchyma was significantly lower in fAIP than in PDAC in all phases. The best diagnostic performance of the rCT value was found in the pancreatic phase, with an area under the ROC curve of 0.912, while the areas under the ROC curve of the portal and delayed phases were 0.812 and 0.754, respectively. The optimal cut‐off value for distinguishing fAIP from PDAC was 1.62 in the pancreatic phase.

**Conclusions:**

The rCT of the pancreatic parenchyma during the pancreatic phase may be a feasible CT feature for differentiating fAIP from PDAC.

## Graphical abstract

Similar imaging features such as regional enlargement of the pancreas and delayed enhancement became a big challenge to differentiate focal‐type autoimmune pancreatitis (fAIP) from PDAC. In this study, we found that the pancreatic parenchyma of fAIP presented severe inflammation. Moreover, the mean relative computed tomography values of the pancreatic parenchyma were lower in fAIP than that in PDAC in all phases.

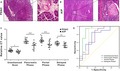

## 1 INTRODUCTION

Autoimmune pancreatitis (AIP) is a unique form of pancreatitis with abundant pathological lymphoplasmacytic infiltration pathologically.^1^, ^2^ Recent studies have classified AIP into two groups: the diffuse and focal subtypes.^3^, ^4^ focal‐type autoimmune pancreatitis (fAIP), accounting for approximately 33%‐41% of all cases, shares similar features with pancreatic ductal adenocarcinoma (PDAC); the features include focal or mass‐like enlargement of the pancreas and obstructive jaundice.^3^, ^5^, ^6^ However, the treatment of fAIP and PDAC is completely different. Corticosteroid therapy is an effective treatment for fAIP,^7^ while surgery, chemotherapy and radiotherapy are often used in PDAC treatment. Thus, accurate diagnosis of fAIP and PDAC plays an important role in their management. Disappointingly, similar imaging features such as regional enlargement of the pancreas and delayed enhancement cause challenges in differentiating fAIP from PDAC.^4^, ^8^, ^9^, ^10^, ^11^ An estimated 3%‐9% of fAIP patients have been reported to undergo resection for a presumed carcinoma.^12^ Thus, accurate diagnosis of fAIP vs PDAC is critical.

Recent studies have shown that various diagnostic imaging findings are useful in differentiating fAIP from PDAC,^10^, ^13^, ^14^, ^15^ such as a mass showing homogeneous enhancement during the portal phase, a capsule‐like rim, a duct‐penetrating sign and an enhanced duct sign. However, the accuracy of the diagnosis based on previous reports is unsatisfactory.^10^, ^14^, ^16^, ^17^ Therefore, signs with higher sensitivity are necessary for the differentiation of fAIP from PDAC.

Considering that fAIP is a systemic immune disease with lymphoplasmacytic infiltration and the proliferation of fibrous tissue, we speculated that the adjacent pancreatic parenchyma of the fAIP might also show hyperplastic fiber tissue with the infiltration of chronic inflammation cells, which would differ from that of PDAC. This histological difference between fAIP and PDAC may be reflected on imaging. Thus, our study retrospectively analyzed the enhanced computed tomography (CT) features of adjacent pancreatic parenchyma in fAIP and PDAC patients and evaluated whether the enhancement of the adjacent pancreatic parenchyma of the two conditions could differentiate fAIP from PDAC.

## 2 MATERIALS AND METHODS

### 2.1 Patients

Diagnosis of fAIP was made on the basis of the following three items: (a) radiological imaging showing regional enlargement of the pancreas; (b) laboratory data showing abnormally elevated levels of serum γ‐globulin or immunoglobulin G (IgG) or the presence of autoantibodies; and (c) histological examination of the pancreas showing lymphoplasmacytic infiltration and fibrosis.

**Figure 1:**
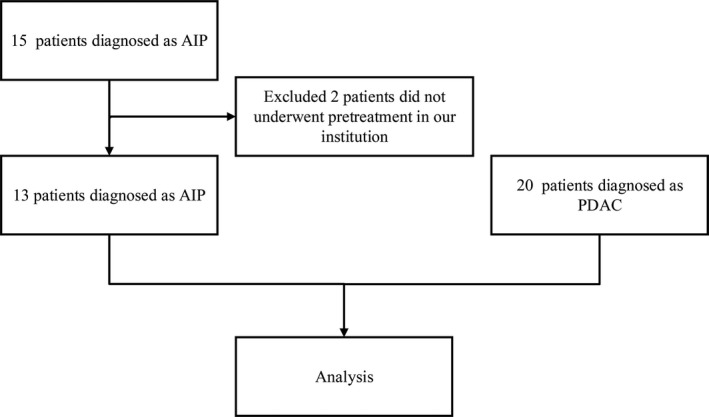
A flow chart of the patient selection process. AIP, autoimmune pancreatitis; PDAC, pancreatic ductal adenocarcinoma

A total of 15 patients were pathologically diagnosed with fAIP between January 2013 and December 2016 at our institution. Those who did not undergo a pretreatment CT examination in our department were excluded (n = 2). Finally, 13 patients diagnosed with fAIP were enrolled in this study. Additionally, a total of 20 patients pathologically diagnosed with PDAC (histopathological examination of the sample from resection) between January 2016 and December 2016 were also enrolled in this study. The flow chart of the patient selection process is shown in Figure 1. This study was approved by the Institutional Review Board (IRB) of the Sun Yat‐sen University Cancer Center. All procedures performed in studies involving human participants were conducted in accordance with the ethical standards of the institutional and/or national research committee and with the 1964 Helsinki Declaration and its later amendments or comparable ethical standards. Written informed consent was obtained from patients prior to treatment.

### 2.2 CT imaging protocol

Computed tomography imaging was obtained from a 64‐slice spiral CT system (Aquilion TSX‐101A; Toshiba Medical System), a 128‐slice spiral CT system (Discovery CT750 HD; GE System), a 256‐slice spiral CT system (Brilliance iCT; Philips System) or a dual‐source spiral CT system (SOMATOM Force; Siemens Medical System). Computed tomography studies were performed using the following parameters: slice thickness, 5 mm; slice interval, 1 mm; tube voltage, 80‐140 kVp; tube current, automatic tube current modulation (maximum 450) mAs; and sagittal and coronal reconstruction thicknesses, 2 mm with 2‐mm intervals.

After unenhanced images had been acquired, all patients underwent pancreatic, portal and delayed phase imaging. The average scan delays from the injection of contrast material to the start of pancreatic, portal and delayed phase imaging were 38, 60 and 180 seconds, respectively. Contrast‐enhanced CT was performed after an intravenous bolus dose of 1.5‐2 mL/kg body weight of a nonionic iodinated contrast agent (iopromide; Ultraist 300; Schering) that was administered into the antecubital vein at a rate of 3.0 mL/s via a high‐pressure syringe.

### 2.3 CT image analysis

Two radiologists with 15 and 8 years of experience in the interpretation of abdominal imaging independently reviewed the CT images for each patient. They were blinded to the final diagnosis and other examination findings. In equivocal cases, discussion occurred until a final consensus was reached.

**Figure 2:**
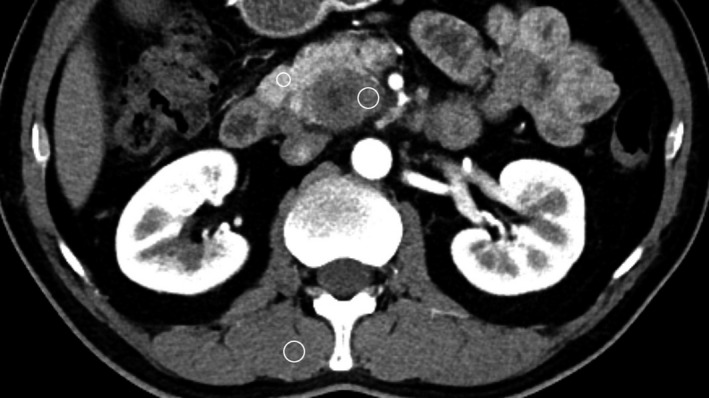
The axial enhancement computed tomography image shows a focal lesion in the pancreatic head that presented as a decreased enhanced area during the pancreatic phase. A spherical region of interest (ROI) was placed in the enhanced part of the lesion, avoiding suspected necrotic regions. A spherical ROI was placed in the adjacent pancreatic parenchyma within 10 millimeters of the lesion, avoiding the pancreatic lesion and pancreatic duct, and the partial volume was averaged for the extrapancreatic structures. A spherical ROI was placed in the erector spinae muscle

Computed tomography attenuation values were measured by two radiologists using a workstation (Advantage version 4.2; GE Healthcare). Computed tomography attenuation values were measured on unenhanced images of the pancreatic and portal and delayed phases after contrast administration. (a) The CT attenuation value of the adjacent pancreatic parenchyma was measured by the placement of a region of interest (ROI) in the pancreatic parenchyma within 10 mm of the lesion, avoiding the pancreatic mass; the pancreatic duct and partial volume were averaged from the extrapancreatic structures. The spherical ROI (approximately 5‐10 mm in diameter) was placed in three segments of the adjacent pancreatic parenchyma, and the mean value was calculated. (b) Computed tomography attenuation values of the lesion were measured by the placement of a spherical ROI in three enhanced segments of the lesion, and the mean value was calculated. The ROI was placed to avoid suspected necrotic regions or the calcification of vessels or ducts to minimize the influence of potential measurement errors. (c) Computed tomography attenuation values of the muscle were similarly measured by placing a spherical ROI in the erector spinae muscles thrice three times, and the mean value was calculated. The ROI was approximately 10 mm in diameter. Figure 2 indicates the ROI placement and the measurement of the CT attenuation value.

Delayed enhancement of a mass or segment was defined as a greater than 15­HU increase in the CT attenuation of the pancreatic mass between the pancreatic phase and the portal phase. A relative CT attenuation value (rCT) of the adjacent pancreatic parenchyma was calculated as the ratio of the CT value of the adjacent pancreatic parenchyma and that of the muscle in the same slice.

**Figure 3:**
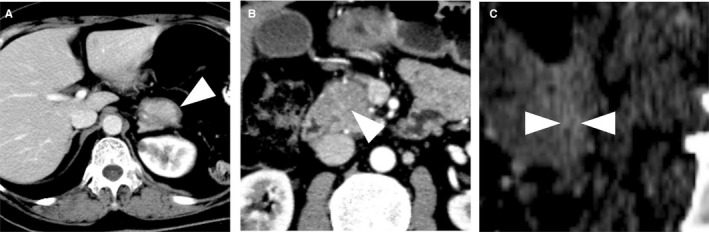
A, A capsule‐like rim is shown as a hypo‐attenuating rim in the surrounding adipose tissue in the axial enhanced computed tomography (CT) images (arrow). B, A “duct‐penetrating sign” can be detected within the lesion presenting as the lumen of the main pancreatic duct (MPD) in the axial CT images (arrow). C, An “enhanced duct sign” is observed in the coronal reconstructed enhanced CT image, showing an enhancement of the MPD wall in the lesion (arrow)

Additionally, other imaging features were defined as follows: (a) homogeneity (homogeneous or heterogeneous); (b) capsule‐like rim (Figure 3A): a low‐attenuation rim surrounding adipose tissue; (c) duct‐penetrating sign (Figure 3B): the detection of the main pancreatic duct (MPD) lumen in the lesions and, if the MPD lumen was detected in the lesions, the length of the visible lumen in the affected area was noted as more than half or less than half; and (d) enhanced duct sign (Figure 3C): the wall enhancement of MPD in the lesion.

### 2.4 Statistical analyses

For continuous variables, the data are expressed as the mean ± SD and were compared using Student's *t* test or paired *t* test (two‐sided). Receiver operating characteristic (ROC) curves were used to evaluate the diagnostic performance of the rCT in different phases. The maximum Youden index was used to identify the best cut‐off point for the CT attenuation value. SPSS (version 21.0; SPSS Inc) was used for statistical analysis. *P* < .05 was considered statistically significant. Mean data are expressed as the means ± SD with their ranges in brackets.

## 3 RESULTS

### 3.1 Patient characteristics

Thirty‐three consecutive patients were enrolled in this study: 10 men and three women (mean age, 56 years; median, 60 years; range, 33‐67 years) with fAIP and 13 men and seven women (mean age, 61 years; median, 57 years; range, 36‐84 years) with PDAC. Table 1 shows the clinical characteristics of the fAIP and PDAC patients. The site of the lesion was the head of the pancreas (12 patients with fAIP and 16 patients with PDAC) or the body or tail of the pancreas (one patient with fAIP and four patients with PDAC). Four of the 13 (31%) patients with fAIP and 12 (60%) of the 20 patients with PDAC were presented with jaundice at diagnosis. The level of the tumor marker carbohydrate antigen 19‐9 was elevated in six (46%) of the 13 patients with fAIP and 16 (80%) of the 20 patients with PDAC according to a cut‐off value of 37 U/mL. Sixteen patients with PDAC in the head and neck of the pancreas underwent pancreaticoduodenectomy, except for four who underwent regional excision of the body and tail of the pancreas. In addition, 12 patients with fAIP in the head and neck of the pancreas underwent pancreaticoduodenectomy, except for one who underwent regional excision of the body and tail of the pancreas.

**Table 1:** Clinicopathological characteristics of patients with PDAC and AIP

Variables	Histological type		*P* value
	PDAC	AIP	
Age			
<60	11	6	.728
≥60	9	7	
Gender			
Female	13	10	.701
Male	7	3	
Tumor location			
Head and neck	16	12	.625
Body and tail	4	1	
Tumor size			
<3 cm	9	6	1.000
≥3 cm	11	7	
CA19‐9			
<37 U/mL	4	7	.065
≥37 U/mL	16	6	

Abbreviations: AIP, autoimmune pancreatitis; CA19‐9, Carbohydrate antigen19‐9; PDAC, pancreatic ductal adenocarcinoma.

### 3.2 Both fAIP and PDAC presented hyper‐fibrosis histologically and delayed enhancement on CT examination

**Figure 4:**
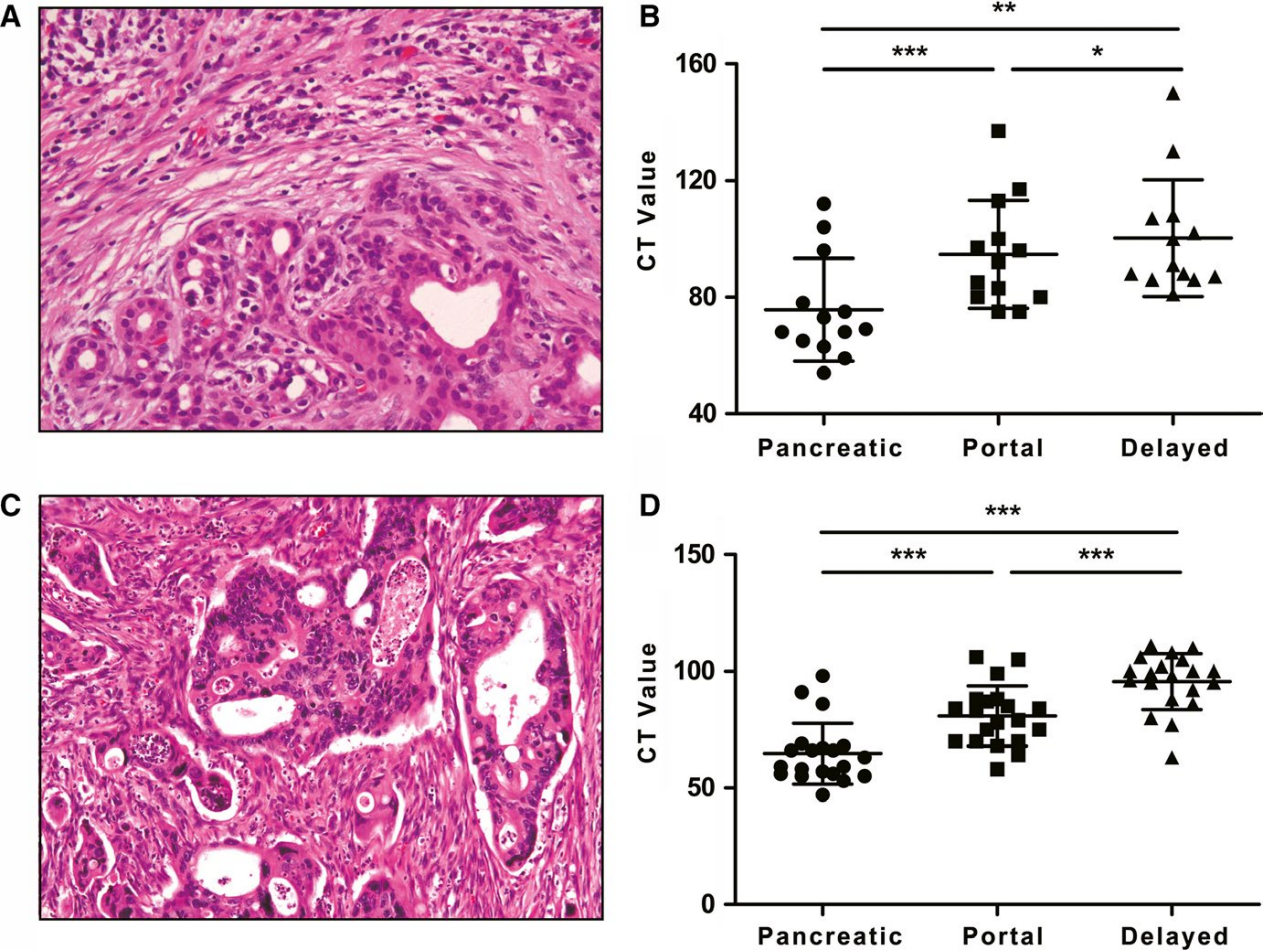
A, Hematoxylin‐eosin staining revealed adjacent pancreatic parenchyma fibrous tissue proliferation with prominent chronic inflammation and normal pancreatic ducts. B, Autoimmune pancreatitis presented delayed enhancement on computed tomography (CT) scan. The CT value increased with the phase. (*P <.05 and >.01, **P <.01 and >.001, ***P < .001). C, Hematoxylin‐eosin staining revealed fibrous tissue proliferation, desmoplasia and irregular gland formation, and the cells demonstrated marked cytological atypia. D, Pancreatic ductal adenocarcinoma presented delayed enhancement on the CT scan. The CT value increased with the phase. (*P <.05 and >.01, **P <.01 and >.001, ***P < .001)

**Figure 5:**
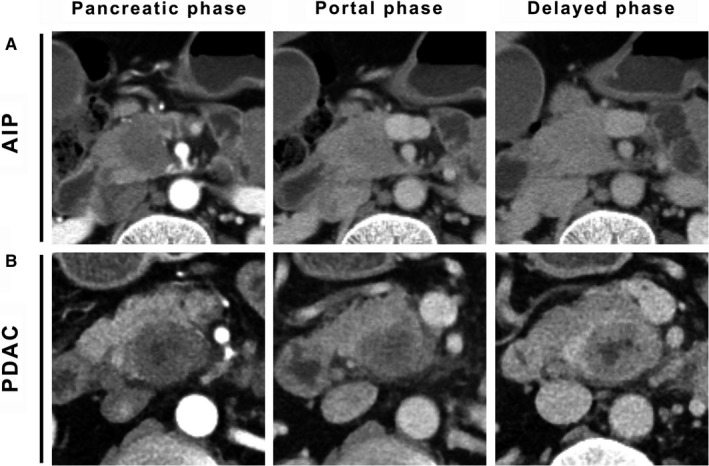
A, A focal lesion shows homogeneously decreased enhancement during the pancreatic phase and homogeneously delayed enhancement during the portal and delayed phases. B, The tumor shows a heterogeneously decreased enhancement during the pancreatic phase and delayed enhancement during the delayed phase. There is a patchy area in the central part of the tumor that shows no enhancement during the pancreatic, portal or delayed phase. AIP, autoimmune pancreatitis; PDAC, pancreatic ductal adenocarcinoma

First, as shown in Figure 4A,C, we found that both fAIP and PDAC presented hyper‐fibrosis on postoperative pathology. Next, CT analysis was performed on the pretreatment CT for the 13 fAIP patients and 20 PDAC patients. As shown in Figure 4B,D, both fAIP and PDAC presented with hypo‐attenuation during the pancreatic phase and iso‐attenuation or hyper‐attenuation during the portal or delayed phase, showing a delayed enhancement pattern. Figure 5 shows representative images of the pancreatic, portal and delayed phases in patients with AIP and PDAC.

### 3.3 Pathological characteristics of the adjacent pancreatic parenchyma in fAIP and PDAC

**Figure 6:**
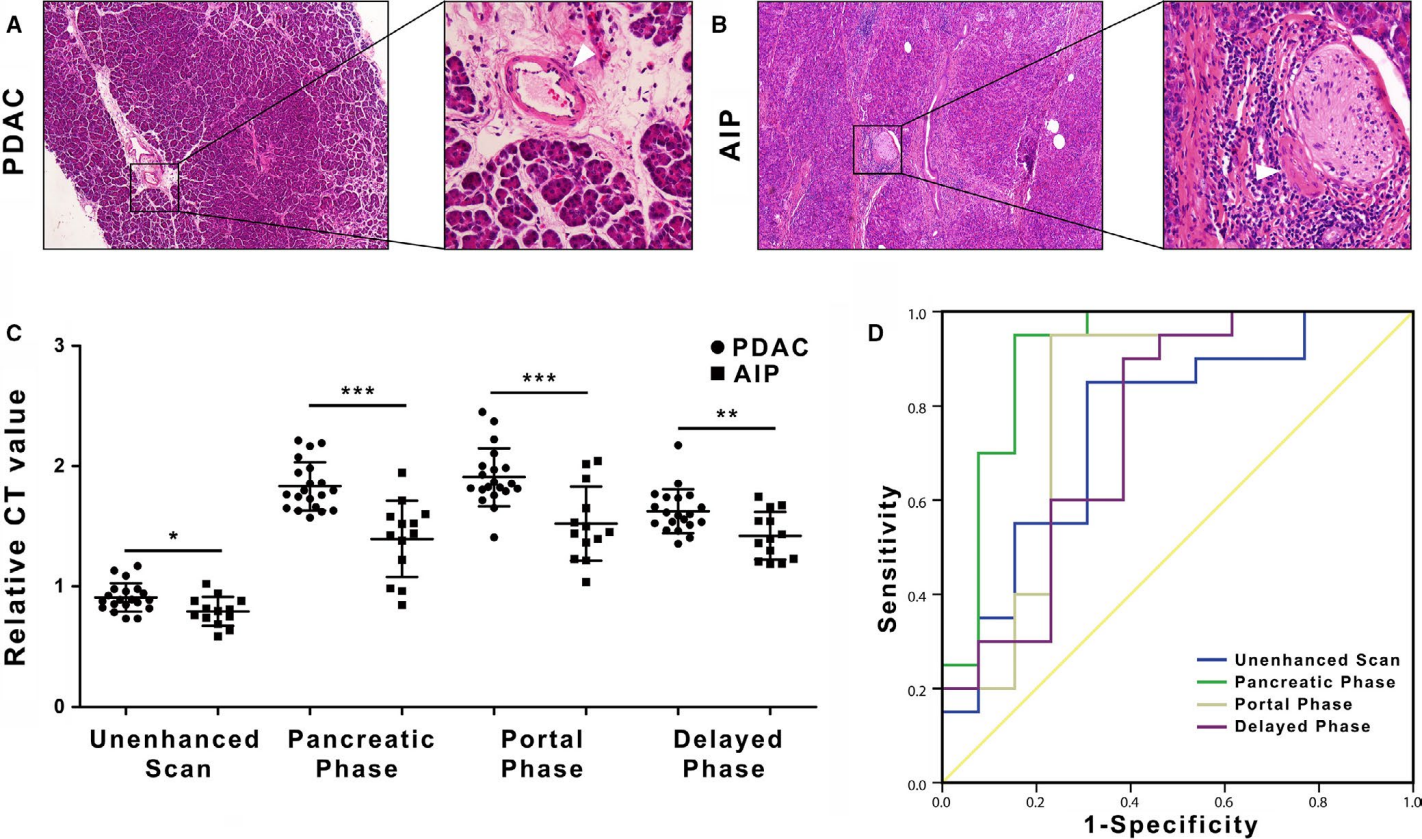
A, The histological characteristics of the adjacent para‐carcinoma pancreatic tissues of autoimmune pancreatitis (AIP) include fibrous proliferation of interlobular portal areas, and the lobular structure is not clear. At high magnification, abundant lymphoplasmacytic infiltration and small vessel occlusion can be observed. B, The adjacent para‐carcinoma pancreatic tissues of pancreatic ductal adenocarcinoma (PDAC) appear normal with a clear lobular structure, normal small vessels and a few inflammatory cells. C, Computed tomography (CT) attenuation value of the adjacent pancreatic parenchyma was lower in focal‐type autoimmune pancreatitis (fAIP) than in PDAC. The CT attenuation values of the adjacent pancreatic parenchyma in fAIP were significantly lower than those in PDAC in the pancreatic, portal and delayed phases (1.83 vs 1.40, *P* < .001; 1.91 vs 1.52, *P* < .001; 1.63 vs 1.42, *P* = .005, respectively). D, The best diagnostic performance of relative CT was found in the pancreatic phase, with an area under the receiver operating characteristic (ROC) curve of 0.912, while the area under the ROC curve values of the portal and delayed phase were 0.812 and 0.754, respectively. The optimal cut‐off value for distinguishing between fAIP and PDAC was 1.62 in the pancreatic phase. *P < .05; **P < .01; ***P < .001

As shown in Figure 6A, the pancreatic parenchyma adjacent to fAIP also presented fibrous proliferation of the interlobular portal areas, an unclear lobular structure, a relatively high quantity of infiltrated lymphocytes and plasmocytes, and small vessel occlusion. In contrast, as shown in Figure 6B, the para‐carcinoma tissue of PDAC presented as normal pancreas, with a clear lobular structure, normal small vessels and few inflammatory cells.

### 3.4 rCT of the adjacent pancreatic parenchyma in fAIP and PDAC

As shown in Table 2, the rCT values in the adjacent pancreatic parenchyma in patients with fAIP were significantly lower than those in patients with PDAC in the pancreatic, portal and delayed phases (1.83 vs 1.40, *P* < .001; 1.91 vs 1.52, *P* < .001; 1.63 vs 1.42, *P* = .005, respectively) (Figure 6C).

Moreover, we found that the CT attenuation value in the adjacent pancreatic phase had the highest area under the curve (AUC) value (0.912), while those of the portal and delayed phases were 0.812 and 0.754, respectively (Figure 6D).

### 3.5 Other characteristic imaging findings in fAIP

This study identified homogeneous good enhancement during the portal and delayed phases that was displayed in 10 of the 13 (76%) patients with fAIP. A capsule‐like rim was detected in five of the 13 (38%) patients with fAIP. In this study, the frequency at which the duct‐penetrating sign was observed was six out of the 13 patients with fAIP (46%). In addition, an enhanced duct sign was present in five of the 13 (38%) patients with fAIP. Figures 3 and 5 show the representative images of these features.

## 4 DISCUSSION

This study sought to analyze the enhanced characteristics of adjacent pancreatic parenchyma in fAIP and PDAC, and we found that the CT attenuation value of the adjacent pancreatic parenchyma in the pancreatic phase was significantly lower in patients with PDAC than in those with fAIP. In addition, the diagnostic AUC of the rCT value in the pancreatic phase was 0.912.

Recent studies revealed that both fAIP and PDAC present as delayed enhancements during the portal and delayed phases,^4^, ^8^, ^9^, ^10^, ^11^ which was also observed in our study. Moreover, a recent study showed that abundant fibrous stroma usually resulted in delayed enhancement.^18^ Pathologically, we detected that both fAIP and PDAC presented with abundant fibrous stroma. Thus, the characteristic of delayed enhancement of the tumor could not be used to differentiate between the two conditions.

Considering that fAIP is a part of a systematic immune disease with the infiltration of lymphoplasmacytic cells, the proliferation of fibroblasts, and angiographic abnormalities such as obliterative arteritis and obliterative phlebitis induced by the inflammatory process,^5^, ^19^, ^20^ we speculated that the pancreatic parenchyma adjacent to fAIP is also infiltrated by lymphocytes, plasmocytes and fibrosis, which differs from the situation in PDAC. This histological difference between fAIP and PDAC may be reflected on imaging. In our study, we found that the pancreatic parenchyma adjacent to fAIP presented fibrous proliferation of the interlobular portal areas, an unclear lobular structure, abundant lymphoplasmacytic infiltration and small vessel occlusion, while that adjacent to PDAC presented as normal, with a clear lobular structure, normal small vessels and few inflammatory cells. This observation showed that the pancreatic parenchyma adjacent to fAIP presented with more serious inflammation and a higher degree of fibrosis than that adjacent to PDAC, potentially resulting in less blood flowing into the tissue. As a result, the CT attenuation value of pancreatic parenchyma adjacent to fAIP may be lower than that of pancreatic parenchyma adjacent to PDAC. Consistent with our speculation, the CT attenuation value of the pancreatic parenchyma adjacent to fAIP was significantly lower than that of the pancreatic parenchyma adjacent to PDAC in the pancreatic phase (*P* < .001). Similarly, Takahashi et al^13^ quantitatively assessed dual‐phase contrast‐enhanced CT scans among 43 AIP patients and 25 patients with normal pancreases and found that the mean CT attenuation value of the pancreatic parenchyma in patients with AIP was significantly lower than that in patients with normal pancreases in the pancreatic phase, which is consistent with the findings of our current study. Moreover, a recent study reported that pancreatic perfusion was reduced in patients with AIP but improved after steroid treatment,^21^ which may also reflect hypo‐vascularity pathologically due to the obliterative phlebitis of the pancreatic vessels with prominent lymphoplasmacytic infiltrate and fibrosis. These data indicated that the CT attenuation value of the adjacent pancreatic parenchyma in the pancreatic phase may be a feasible CT feature for differentially diagnosing fAIP and PDAC.

In addition, recent studies proposed other imaging findings, such as homogeneous enhancement during the portal phases, a capsule‐like rim, a duct‐penetrating sign and an enhanced duct sign, which were also useful findings for the differentiation of fAIP from PDAC.^6^, ^10^, ^14^, ^16^, ^17^, ^18^, ^22^, ^23^ These findings were also observed in our study. In detail, homogeneous enhancement is a widely accepted characteristic that is used to discriminate between fAIP and PDAC.^6^, ^14^, ^17^, ^23^ This study showed that the finding of homogeneous good enhancement during the portal and delayed phases was displayed in 10 of the 13 (76%) patients with fAIP, whereas it was only observed in five of the 20 (25%) patients with PDAC. This finding may histopathologically reflect the presence of inflammatory cell invasion or fibrous tissue in fAIP. PDAC presented as a hypovascular tumor with cystic or necrotic components; these components lead to heterogeneity in the tumor. A capsule‐like rim was detected in 40%‐64% of fAIP lesions in previous studies,^6^, ^10^, ^17^, ^23^ which is presumed to represent a fluid collection, a phlegmon, or fibrosis. In our study, a capsule‐like rim was present in five of the 13 (38%) patients with fAIP. Duct‐penetrating signs were reported in 46%‐73% of patients with AIP.^6^, ^17^, ^22^ In this study, the frequency at which the duct‐penetrating sign was observed was six of the 13 (46%) patients with fAIP. This sign may indicate that MPD is narrowed but not obstructed in fAIP, whereas PDAC readily obstructs the MPD. In addition, an enhanced duct sign has been reported to be present in 45%‐73% of patients with AIP.^16^, ^17^, ^18^ In our study, this sign was present in five of the 13 (38%) patients with fAIP.

This study has several limitations. First, the overall sample size was small, mainly because a large number of patients were lost to follow‐up; thus, the final pathological result remains unknown. In addition, as our study is retrospective, the CT protocols were variable (manufacturers and number of detector rows). It is unclear how the variability in the CT protocol affected our results. Furthermore, a validation study was not performed in other institutions; additional, prospective studies are needed to confirm the results of this study.

In conclusion, our study indicated that the mean rCT of the parenchyma was significantly lower in fAIP than in PDAC in all phases while the best diagnostic performance of the rCT value was found in the pancreatic phase. The predictive value of CT attenuation of the adjacent pancreatic parenchyma during the pancreatic phase might be a feasible CT feature to differentially diagnose fAIP and PDAC and could be used routinely in clinical practice.

**Table 2:** Relative CT value of pancreatic parenchyma in patients with PDAC and AIP

Phase	Relative CT value (Hounsfield unit)		*P* value
	PDAC	AIP	
Unenhanced Scan	0.91 ± 0.12	0.79 ± 0.12	.01
Pancreatic Phase	1.83 ± 0.20	1.40 ± 0.32	<.001
Portal Phase	1.91 ± 0.24	1.52 ± 0.31	<.001
Delayed Phase	1.63 ± 0.18	1.42 ± 0.20	.005

Abbreviations: AIP, autoimmune pancreatitis; CT, computed tomography; PDAC, pancreatic ductal adenocarcinoma
